# A compact diffractive sorter for high-resolution demultiplexing of orbital angular momentum beams

**DOI:** 10.1038/s41598-018-28447-1

**Published:** 2018-07-06

**Authors:** Gianluca Ruffato, Marcello Girardi, Michele Massari, Erfan Mafakheri, Bereneice Sephton, Pietro Capaldo, Andrew Forbes, Filippo Romanato

**Affiliations:** 1 Department of Physics and Astronomy ‘G. Galilei’, University of Padova, via Marzolo 8, 35131 Padova, Italy; 2 LaNN, Laboratory for Nanofabrication of Nanodevices, EcamRicert, Corso Stati Uniti 4, 35127 Padova, Italy; 3 School of Physics, University of the Witwatersrand, Private Bag 3, Wits, South Africa; 4 CNR-INFM TASC IOM National Laboratory, S.S. 14 Km 163.5, 34012 Basovizza, Trieste Italy

## Abstract

The design and fabrication of a compact diffractive optical element is presented for the sorting of beams carrying orbital angular momentum (OAM) of light. The sorter combines a conformal mapping transformation with an optical fan-out, performing demultiplexing with unprecedented levels of miniaturization and OAM resolution. Moreover, an innovative configuration is proposed which simplifies alignment procedures and further improves the compactness of the optical device. Samples have been fabricated in the form of phase-only diffractive optics with high-resolution electron-beam lithography (EBL) over a glass substrate. A soft-lithography process has been optimized for fast and cheap replica production of the EBL masters. Optical tests with OAM beams confirm the designed performance, showing excellent efficiency and low cross-talk, with high fidelity even with multiplexed input beams. This work paves the way for practical OAM multiplexing and demultiplexing devices for use in classical and quantum communication.

## Introduction

During the last decade, Space Division Multiplexing (SDM) has experienced an upsurge of research interest, both in academia and industry, as a possible means to address the ever increasing worldwide demand for bandwidth^1^. In particular, an implementation of SDM exploiting a set of mutually orthogonal spatial modes, in the so-called Mode Division Multiplexing (MDM), has been considered both for free-space and guided propagation, in which independent channels are carried by coaxially propagating and spatially overlapping modes with the same frequency, therefore improving the spectral efficiency and information capacity of the optical link, proportionally to the number of modes transmitted. Among all the different families of orthogonal modal basis, beams carrying orbital angular momentum (OAM) of light have been demonstrated to provide promising candidates for MDM in the optical range^2^, both in free-space^3^ and optical fiber propagation^4^. Beams carrying OAM present a characteristic azimuthal phase term, *exp*(*iℓφ*), being *φ* the azimuthal coordinate on a plane orthogonal to the propagation direction, and *ℓ* the orbital angular momentum per photon in units of *ħ*^5,6^. A pivotal stage of an optical link based on OAM-MDM is that of (de)multiplexing, i.e., how to form a collimated bunch of orthogonal OAM modes at the source and how to sort them according to their OAM content at the receiver after propagation.

Various techniques have been described and implemented in order to sort a set of multiplexed beams with different OAM values, including interferometric methods^7^, time-division techniques^8^, integrated silicon photonics^9^, coherent detection^10^, OAM-mode analysers^11,12^, astigmatic-mode converters^13^, transformation optics^14–16^, and rotational Doppler effects^17^. Among all, one of the most effective methods is represented by transformation optics, mapping (conformally) angular momentum to linear momentum. This involves a unitary transformation converting the azimuthal phase gradients of OAM beams into linear phase gradients (tilted beams), which are then mapped to unique spatial positions by means of a Fourier lens^14^. The mapping is executed by two optical elements in sequence: the first performing a *log-pol* coordinate transformation and the second correcting the introduced phase distortion. This method has been widely used as a sorting technique, for example, in recent telecom experiments both in the classical^18^ and quantum^19,20^ regimes.

In its first realization, spatial light modulators (SLMs) were exploited to implement the two elements^14^, which were subsequently replaced by refractive optical components^15,16^ for efficiency reasons. More recently, diffractive versions^21,22^ exhibiting a higher level of compactness and miniaturization have been realised. The same setup has been demonstrated to perform multiplexing, with the two elements in reverse order^22^.

As shown elsewhere^14–16^, a drawback of this demultiplexing technique is represented by the overlap between neighbouring modes, an unavoidable feature of the design, which is detrimental to the inter-channel cross-talk of the communication system. This can be overcome by using a sparse mode space, but at the expense of discarding many channels included in the sorting bandwidth of the system^21,22^. Therefore, the measurement bandwidth of the sorter, which is proportional to the Fresnel number of the optics^16^, should be increased in order to provide a sufficient number of modes after channel selection. This is achievable for instance by either decreasing the focal length or increasing the size of the first element performing optical transformation^21^. On the other hand, applications in optical fibers could prescribe severe limitations to the number of supported OAM modes, i.e. the maximum *ℓ* value, and the selection of non-consecutive OAM values could dramatically reduce the number of available channels.

An alternative solution consists in including a fan-out element^23^, which creates multiple copies extending the phase gradient of the sorted beam, which is focused as before but with a narrower width, thus improving the separation between spots. This reduces the channel overlap without sacrificing modal density, but comes at the cost of increased size and complexity of the system. In its first realization^24,25^, the fan-out element and the corresponding phase-corrector were realised using spatial light modulators (SLMs) and placed following the two-piece *log-polar* optics, for a total of at least four optical elements, plus lenses in-between for the Fourier transform.

The possibility to integrate the two optical operations into a single optic has been recently demonstrated^26^, again with an SLM, thus prohibiting compactness. Therefore, while these solutions are satisfactory for laboratory tests, they are not suitable for industrial implementation in a real-world communication set-up.

**Figure 1:**
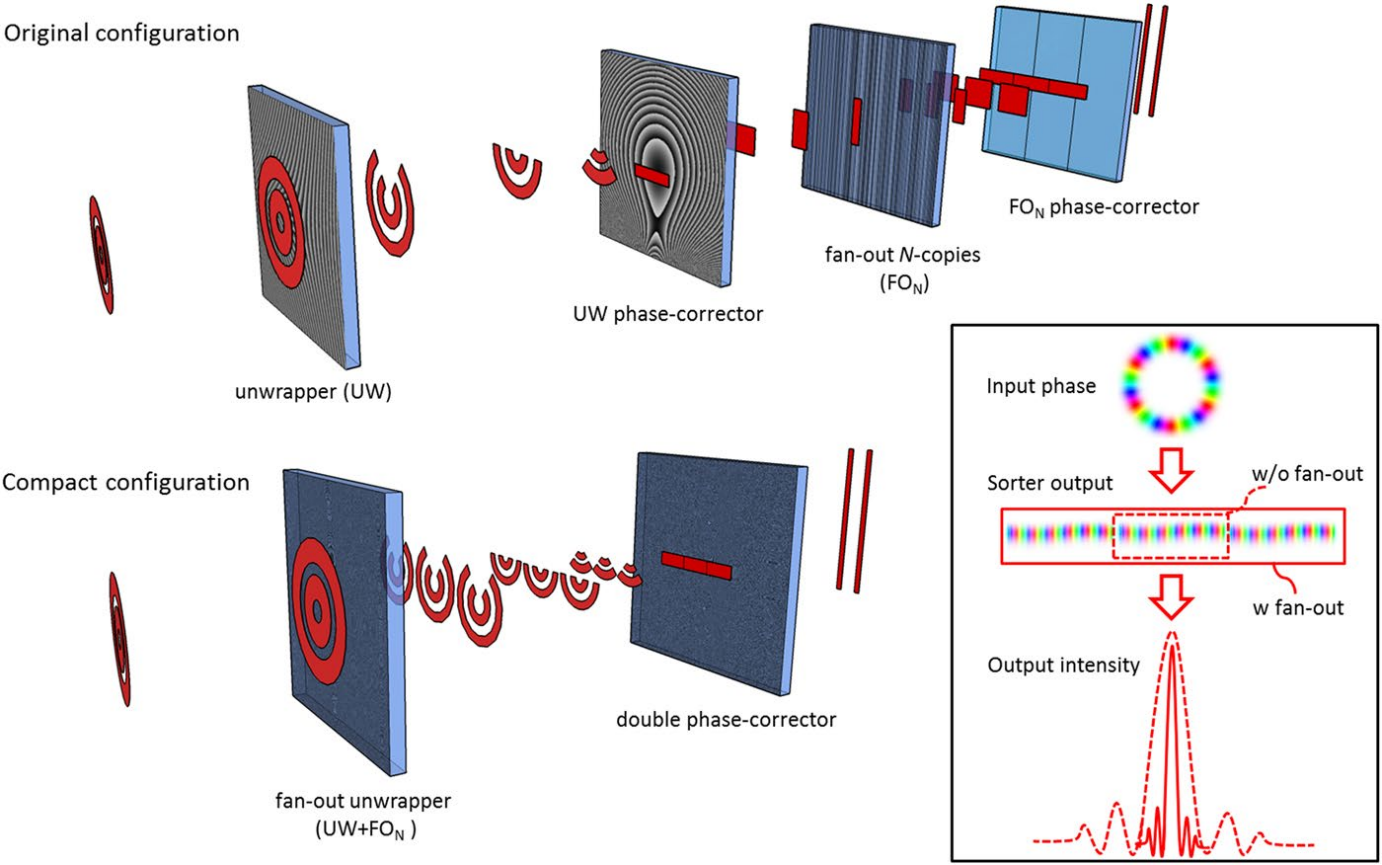
Comparison of high-resolution OAM sorter configurations. In the original configuration, at least four elements were required: an unwrapper and a phase-corrector, followed by a fan-out array and a second phase-corrector, as well as the ubiquitous lenses for Fourier transform. In the new configuration, high-resolution OAM sorting is performed with two diffractive optics: the first encoding both the optical operations of the unwrapper and the fan-out, and the second performing phase-correction. The inset sketches the output intensity and phase with/without (w/o) the fan-out element.

Here we outline a design that allows compactness and efficiency simultaneously, while facilitating ease of production, an important aspect for industrialisation. We fabricate a diffractive solution comprising a first element that performs a *log-pol* optical transformation, fan-out copying and beam focusing, followed by a double phase-corrector which adjusts the phase distortions introduced by the unwrapping and copying processes (Fig. 1). The two optics are integrated into the same optical platform (few mm in thickness) with a single lithographic exposure and constitute a compact high-resolution OAM sorter that simplifies alignment.

## Results

### Sorter design

For the benefit of the reader we briefly summarise the salient points of the design before outlining our compact solution. The optical layout of the sorting system is constituted of two optical elements in sequence: the unwrapper and the phase-corrector. The former performs a conformal mapping of a point (*x*, *y*) in the input plane to a point (*u*, *v*) in the output plane, where *v* = *a* arctan(*y*/*x*) and *u* = −*a* ln(*r*/*b*), being *r* = (*x*^2^ + *y*^2^)^1/2^, *a* and *b* design parameters, while the latter corrects the resultant (distorted) phase by taking into account the optical path differences at each point, thus completing the conversion of the input azimuthal phase gradient into a linear one. After applying the coordinate transformation, the field can be described as a truncated tilted plane wave:

1 $${U}_{\ell }(v)={e}^{i\ell v/a}rect(\frac{v}{2\pi a})$$

where *rect*(*x*) = 1 for |*x*| < 1/2, = 0 otherwise. Therefore, by passing this field through a lens with focal length *f*_T_ and observing the back focal plane, the tilted plane waves angles are converted into lateral shifts Δ*s*_*ℓ*_ proportional to the OAM amount *ℓ* according to:

2 $${\rm{\Delta }}{s}_{\ell }=\frac{\lambda {f}_{T}}{2\pi a}\ell $$

Owing to the non-null width of these spots, there is a significant overlap between the neighbouring modes in the output intensity pattern, which is detrimental when detecting OAM states.

To make the design compact we customized the first element to perform multiple operations at once. The transmission function of this element is then as follows:

3 $${\tau }_{1}(x,\,y)=\exp (i{{\rm{\Omega }}}_{1})=\exp (i{{\rm{\Omega }}}_{UW})\cdot \exp (i{{\rm{\Omega }}}_{FO,N})\cdot \exp (i{{\rm{\Omega }}}_{lens})$$

and results from the combination of three optical elements. The first term performs the *log-pol* optical transformation and is given by^14^

4 $${{\rm{\Omega }}}_{UW}=\frac{2\pi a}{\lambda f}[y\arctan (\frac{y}{x})-x\,\mathrm{ln}(\frac{r}{b})+x]$$

where the two parameters *a* and *b* are related to the optical transformation and control the size and the location of the transformed beam, respectively. The parameter *a* assumes the value *L*/2π, so that the azimuthal phase gradient is mapped over a length *L* on the second element in the *y*-direction, while the position of the unwrapped beam in the *x*-direction is controlled by the parameter *b*.

The second contribution encodes the fan-out term and is given by:

5 $$\exp (i{{\rm{\Omega }}}_{FO,N})=\sum _{m=-(N-1)/2}^{(N-1)/2}{c}_{m}{e}^{i({\gamma }_{m}y+{\delta }_{m})}$$

This term splits the unwrapped beam into *N* copies and locates the several copies of the beam side by side on the second optical element; this is achieved by choosing the spatial frequency carriers according to *γ*_*m*_ = *mLk*/*f*. The parameters (*c*_*m*_, *δ*_*m*_) are optimized for an equal distribution of the input energy over the several copies^27^. After phase-correction, the field results in:

6 $${U}_{\ell }(v)=\frac{1}{\sqrt{N}}{e}^{i\ell v/a}rect(\frac{v}{2\pi aN})$$

which after focusing produces an elongated spot located at the same spacing as before but exhibiting a width scaled as 1/*N*, therefore reducing the overlap between adjacent spots.

Finally, the lens term provides the focusing of the *N*-copies of the unwrapped beam on the second optical element, which phase-corrects the field. This element is a double phase-corrector performing the correction of both the *log-pol* optical transformation and the fan-out process. An analytical formulation of the *log-pol* phase-corrector exists in the paraxial regime, however the particular choices of focal lengths and beam size in this study require a more precise calculation of the phase patterns beyond the Fresnel regime. Based on angular spectrum diffraction theory^28^, the rigorous solution of the diffracted field *U* can be expressed in the convolution algorithm form:

7 $$U(u,\,v)=F{T}^{-1}\{FT\{{\tau }_{1}(x,\,y){U}_{0}(x,\,y)\}{H}_{AS}({f}_{x},\,{f}_{y})\}$$

where *FT* and *FT*^−1^ are the Fourier transform and the inverse Fourier transform, respectively, *H*_AS_ is the angular-spectrum transfer function:

8 $${H}_{AS}({f}_{x},\,{f}_{y})=\exp [ikz\sqrt{1-{(\lambda {f}_{x})}^{2}-{(\lambda {f}_{y})}^{2}}]$$

Then the required phase profile for the phase-correcting term is given by

9 $${{\rm{\Omega }}}_{2}(u,\,v)=2\pi -\arctan [\text{Im}(U)/\mathrm{Re}(U)]$$

This can be calculated numerically for *U*_0_ as an input Gaussian mode with a planar phase front and a beam waist properly chosen in order to illuminate the zone of interest of the first element. In addition, the phase-corrector is endowed with a tilt term to prevent the beam from overlapping with a possible zero-order contribution.

It is well known that traditional OAM sorters are notoriously difficult to align. Moreover, the dual phase-corrector designed in this study requires an even more precise alignment, making it very arduous to obtain output beams of good quality unless the two elements are perfectly planar, coaxial and aligned one to each other. To simplify the alignment process, we designed the sorting configuration in order to incorporate the two elements onto a single optical element.

**Figure 2:**
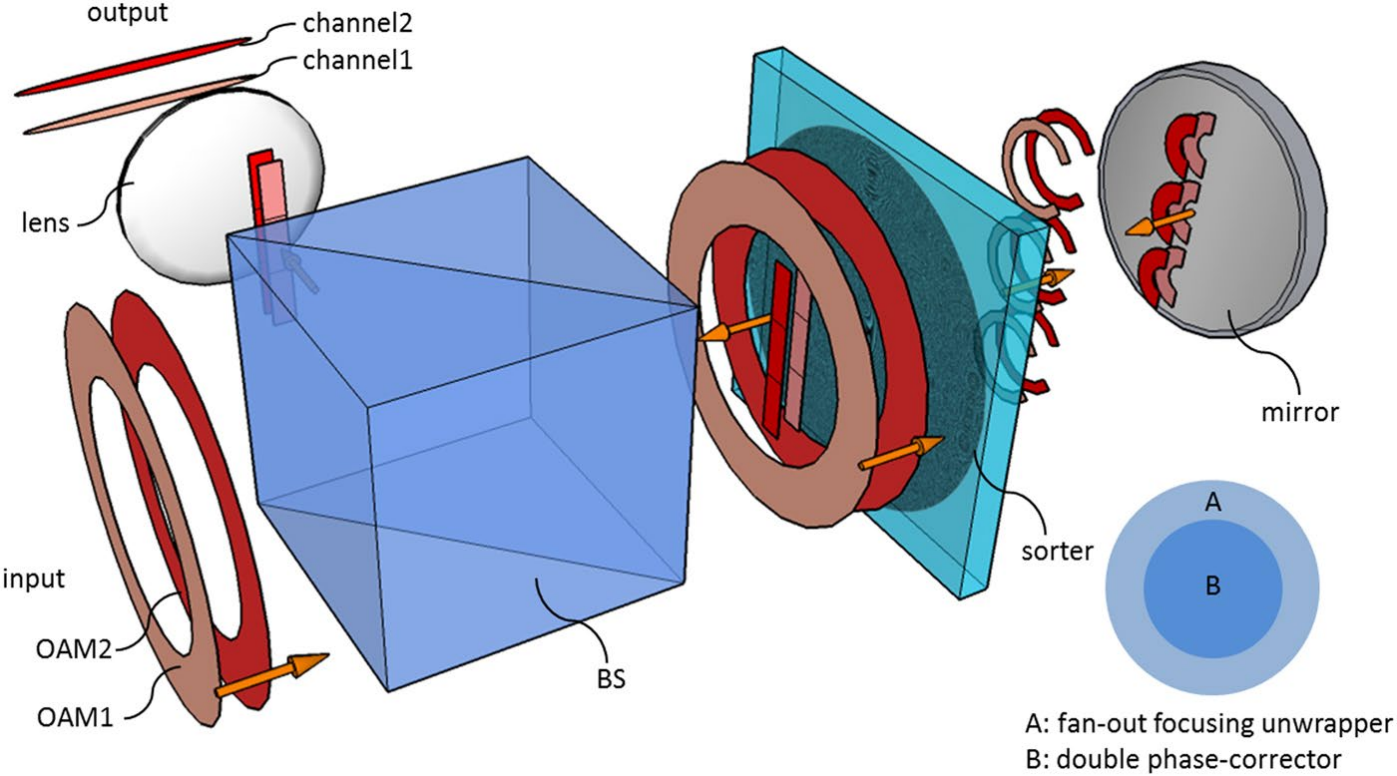
Schematic of the compact high-resolution sorter. The input OAM beam impinges on the outer annular region (**A**) of the diffractive optics that encodes the fan-out focusing unwrapper. Subsequently, a mirror reflects the beam towards the optical element again through its inner zone (**B**), where double phase-correction is performed. A Fourier lens completes the demultiplexing. A beam-splitter (BS) is used to separate input and output beams.

**Figure 3:**
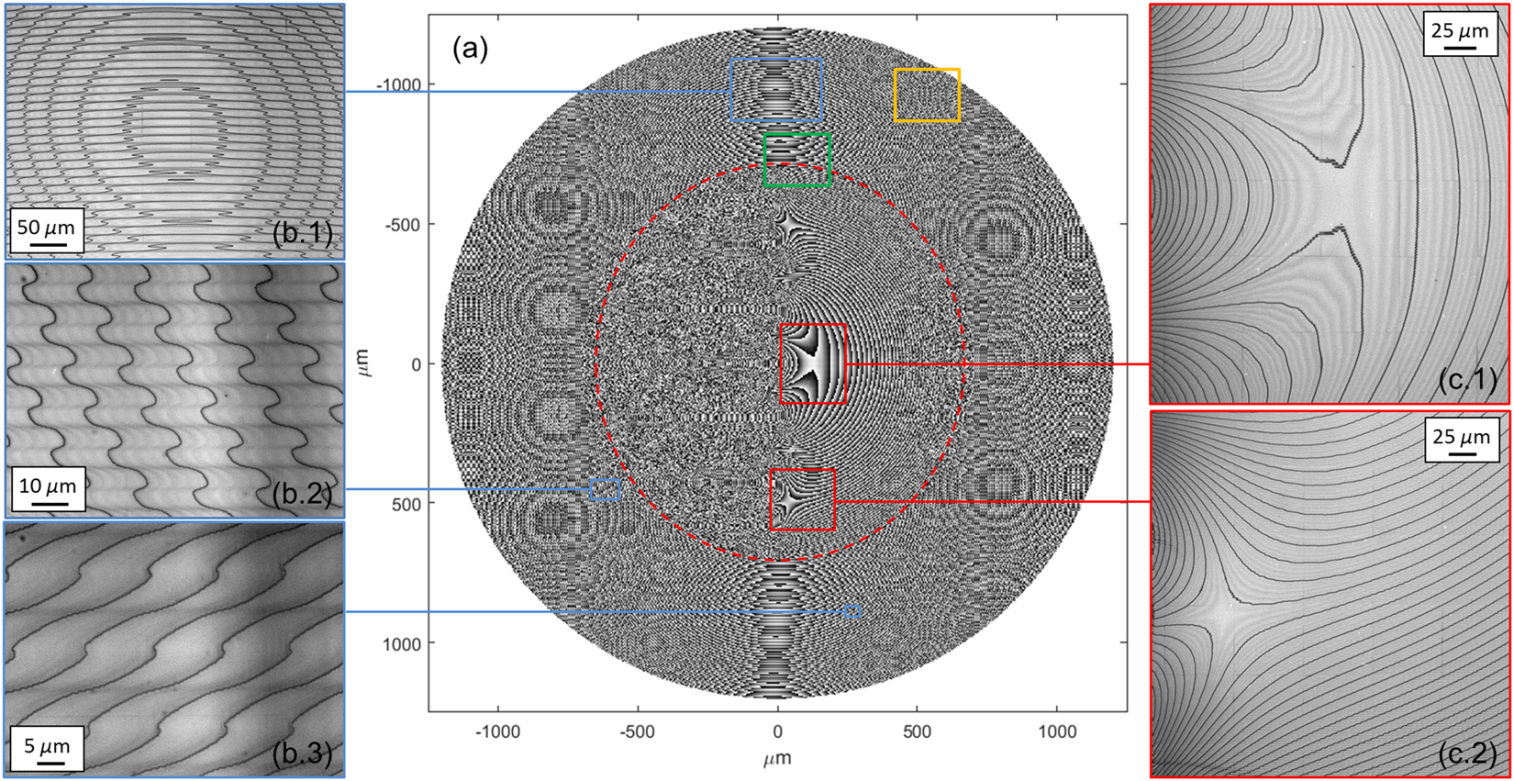
(**a**) Phase pattern of *log-pol* sorter with three-copies fan-out. Parameters of the optical transformation: *a* = 64 μm, *b* = 900 μm, *f* = 8.5 mm, phase-corrector radius (red dashed line) *ρ*_2_ = 700 μm, unwrapper outer radius *ρ*_1_ = 1200 μm. (**b.1-3**) Details of the fan-out unwrapper zone at optical microscope. (**c.1-2**) Details of the double phase-corrector region at optical microscope.

The integration hinges on the fact that OAM beams have a doughnut-like intensity profile around a central null. Since the optical element acts basically on the zone with non-zero input field, the first transformation leaves unexploited the inner region of the optics. Therefore, this central zone can be selected for integrating the second element performing phase-correction. In this novel configuration, the optical element is illuminated twice: after crossing the outer unwrapping zone, the beam is back-reflected with a mirror and impinges on the inner central region providing the phase-corrector (see Fig. 2). Therefore, the total diffractive phase pattern Ω_DOE_ turns out to be the composition of the two phase functions in Eqs (3) and (9):

10 $${{\rm{\Omega }}}_{DOE}={{\rm{\Omega }}}_{1}{\rm{\Theta }}(\rho -{\rho }_{2}){\rm{\Theta }}({\rho }_{1}-\rho )+{{\rm{\Omega }}}_{2}{\rm{\Theta }}({\rho }_{2}-\rho )$$

being *ρ*_1_ the outer radius of the optics, *ρ*_2_ the radius of the central part, Θ the Heaviside function (Θ(*x*) = 1 for *x* > 0, Θ(*x*) = 0 otherwise), providing the condition *ρ*_2_ > *N*π*a* is fulfilled. This solution makes the alignment operation significantly easier, since the two elements are by-design aligned, parallel and coaxial one to each other. However, this imposes limitations on the input-beam radius and on the lateral extension of the phase-corrector, i.e. the number of copies *N*. Figure 3(a) shows the phase pattern calculated for the following optical-transformation parameters: *a* = 64 μm, *b* = 900 μm, *f* = 8.5 mm, three-copies fan-out. The outer radius is equal to 1200 μm. In order to satisfy the above restriction *ρ*_2_ > 3π*a* ( = 603.2 μm), we chose the value *ρ*_2_ = 700 μm.

### Electron-beam lithography

The designed fan-out sorters were fabricated as surface-relief phase-only diffractive optics. Electron-beam lithography is known to be the best technique to fabricate 3D profiles with nanometric resolution^29^. By locally controlling the released electronic dose, a different dissolution rate is induced in each zone of the exposed polymer, giving rise to different resist thicknesses after the development process. A dose-depth correlation curve (contrast curve) is required to establish the correct electron-dose to assign to each zone in order to obtain the desired resist thickness. By using custom numerical codes, the transmission phase function of the simulated optics was converted into a 3D multilevel structure, whose local thickness was proportional to the theoretical phase delay, which was in turn transformed into a map of electronic doses. A dose correction for compensating the proximity effects was applied, in order both to match layout depth with the fabricated relief and to obtain a good shape definition, especially in correspondence of the phase discontinuities. In this work, the diffractive-optics phase patterns were written on a PMMA resist layer with a JBX-6300FS JEOL EBL machine, at 5 nm resolution, working at 100 KeV with a current of 100 pA, using a proximity-effect correction assisted ultra-fine process. The substrate used for fabrication was glass-coated ITO with low surface resistivity (8–12 Ω) in order to ensure a good discharge of the sample during electron beam lithography. After the exposure, the resist was developed in a temperature-controlled developer bath for 60 s.

At the experimental wavelength of the laser (λ = 632.8 nm), the PMMA refractive index was assessed to be *n*_PMMA_ = 1.489, as measured by analysis with a spectroscopic ellipsometer (J.A. Woollam VASE, 0.3 nm spectral resolution, 0.005° angular resolution). For a phase pattern of Ω(*x*, *y*), the depth *t*(*x*, *y*) of the exposed zone for normal incidence in air is given by:

11 $$t(x,\,y)=\frac{\lambda }{{n}_{PMMA}-1}\cdot \frac{2\pi -{\rm{\Omega }}(x,\,y)}{2\pi }$$

**Figure 4:**
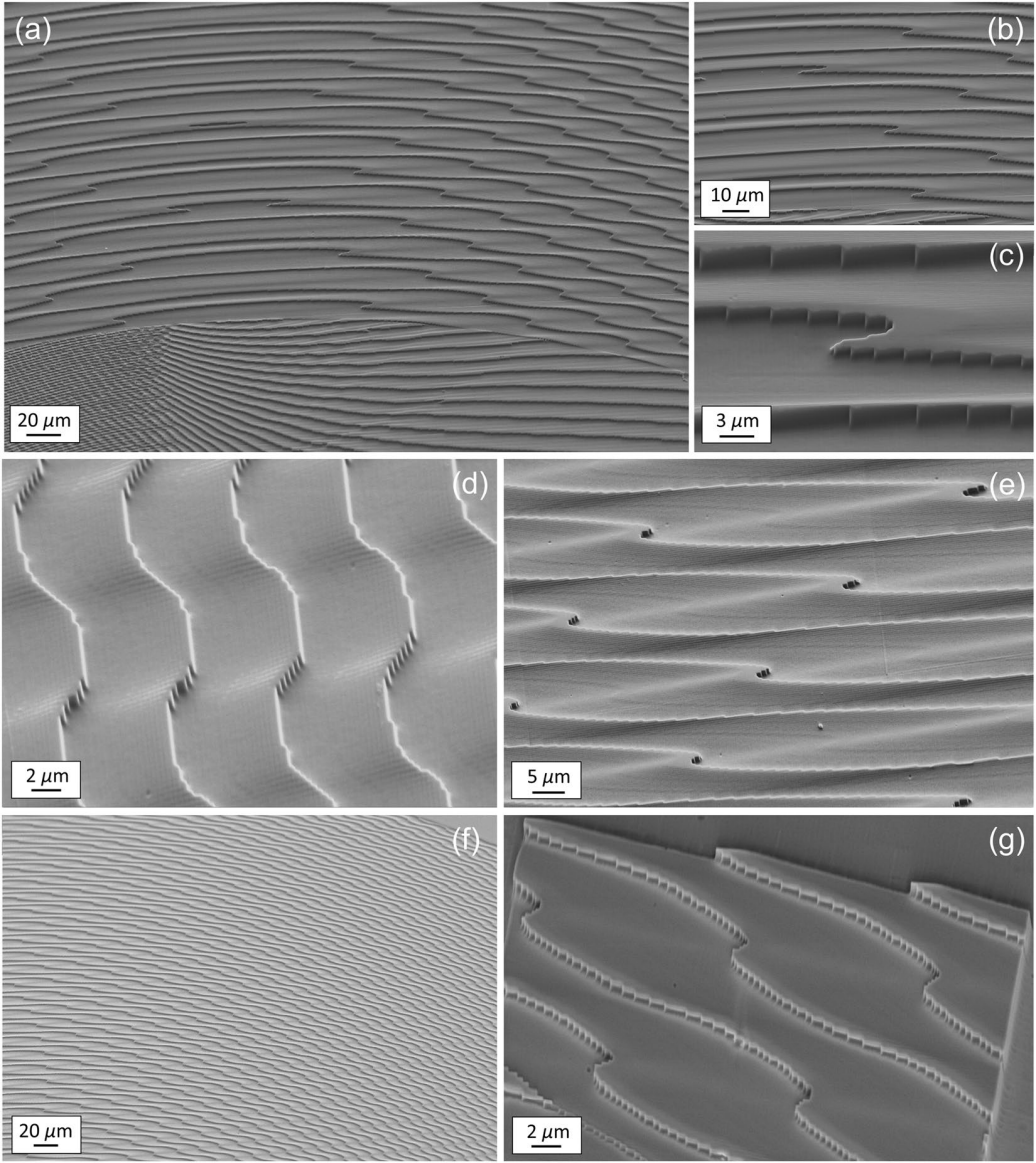
SEM inspection of the 3-copy sorter fabricated with electron-beam lithography and designed for *λ* = 632.8 nm. Number of phase levels: 256. Maximal thickness step: 1289.0 nm. (**a**) Zone of transition from the inner (double phase-corrector) to the outer (fan-out unwrapper) region marked by a green line in Fig. 3(a). (**b**–**e**) Details. (**f**) Region marked by an orange line in Fig. 3(a). (**g**) Exposure of a limited part (40 × 40 μm^2^) of the region in figure (**f**) in order to highlight the edge profile.

The fabricated optics were made of pixels square matrices with *M* = 256 phase levels. Each pixel area measures 0.312 × 0.312 μm^2^. Inserting the values of the laser wavelength and PMMA refractive index into the previous equation, the maximal depth of the surface relief pattern was found to be 1289.0 nm, with a thickness resolution of Δ*t = *5.1 nm. The quality of the fabricated samples was assessed with optical (Fig. 3) and scanning-electron microscopy (Fig. 4).

A soft-lithography replica process of the PMMA masters was fine-tuned and optimized in order to set-up an easy, fast and low-cost fabrication protocol. The optical characterization of the generated copies has demonstrated exceptional fidelity in replicating the 3D structure of the diffractive optics.

### Optical characterization

Optical vortices were generated by illuminating a computer-controlled LCoS spatial light modulator (PLUTO-NIR-010-A, Holoeye, 1920 × 1080 pixels, 8 μm pixel size, 8-bit depth) with a linearly polarized Gaussian beam (*λ* = 632.8 nm, beam waist *w*_0_ = 240 μm, power 0.8 mW) emitted by a HeNe laser source. The following phase pattern Ω_SLM_ was uploaded on the SLM display, combining spiral phase and axicon functions with a quadratic contribution for curvature correction:

12 $${{\rm{\Omega }}}_{SLM}(r,\,\phi )=\ell \phi -\alpha r-k\frac{{r}^{2}}{2R}$$

being *α* the axicon parameter, *k* = 2*π/λ* the wavevector in air, and *R* the curvature radius of the beam illuminating the display. As outlined in^30^, a high-order Bessel Gaussian beam is created in the far field, which can be transformed by a Fourier lens into an annular ring, whose radius and width do not depend significantly on the carried OAM and can be controlled by changing the axicon parameter and the input beam-waist. This solution allows limiting the input ring distribution of the OAM beam intensity to the outer region of the optics, encoding the fan-out unwrapper, irrespective of the OAM content for any value of *ℓ*, *ℓ* = 0 included. In the experimental setup, a first lens of focal length *f*_1_ = 40 cm was used to Fourier-transform the beam reflected by the SLM. At the back focal plane of this lens, a ring was formed having a diameter of *R*_*V*_ ≃ *αf*_1_/*k*, topological charge *ℓ* and a width Δ*R*_V_ = 2*f*_1_/(*kw*), being *w* the beam-waist incident on the SLM. With the choice *α* = 0.22 and *w* = 873 μm, the OAM beams exhibit ring radius and width around 900 μm and 100 μm, respectively, and well fit the outer annular zone of the optics, which is limited between 700 μm and 1200 μm (see Fig. 3). A 50:50 beam-splitter was used to split the beam and analyze the field profile with a CMOS camera. Next, the generated perfect vortex illuminated the first outer zone of the fan-out sorter, mounted on a 6-axis kinematic mount and placed at the focus of the lens. The transmitted beam was back-reflected by a mirror fixed on a kinematic mount, at a distance equal to half the focal length *f* of the unwrapping term in Eq. (4), finely adjustable with a micrometric translator. After illuminating the sorter inner zone, the signal was collected by a second CCD placed at the back-focal plane of a second lens with *f*_2_ = 12.5 cm.

The optical performance of the fabricated compact sorters was characterized by illuminating, in sequence, with beams carrying OAM in the range from −9 to +9 and recording the intensity profiles.

The area of the CCD was divided into rectangular regions of interest, with the center on each elongated spot in far-field, and a width given by the minimum distance between any two adjacent channels. By integrating the total intensity in each region of interest, the relative modal power and modal cross-talk into neighbouring channels could be determined. The cross-talk *XT*_*j*_ on the *j*th channel was calculated using the following definition:

13 $$X{T}_{j}=10\cdot {\mathrm{log}}_{10}(\frac{{I}_{j,ALL\backslash j}}{{I}_{j,ALL}})$$

being *I*_*j,ALL*_ the signal at the *j*th channel when all the input channels were on, *j* included, while *I*_*ALL\j*_ was the same measure when the *j*th input channel was off.

Then, by utilizing a phase and amplitude modulation technique^31^ on the SLM, it was additionally possible to study the performance of the sorters with regards to sorting multiplexed OAM modes. Weighted superposition of Laguerre-Gaussian (LG) modes were generated in the first diffraction order of the SLM and imaged through a *f-f* lens configuration onto the sorter system concatenation of fan-out *log-pol* elements and the transforming lens. To study the accuracy of multimode beam detection with LG beams, we considered the fan-out *log-pol* sorter formed by the two elements separated, in sequence, as shown in the Supplementary Fig. 3. Detection and comparison of the relative intensities in the associated CCD bins to the weightings encoded on the hologram for adjacent OAM values provided an indication of the accuracy in demultiplexing multimode OAM beams, i.e., beams comprising several OAM values simultaneously.

**Figure 5:**
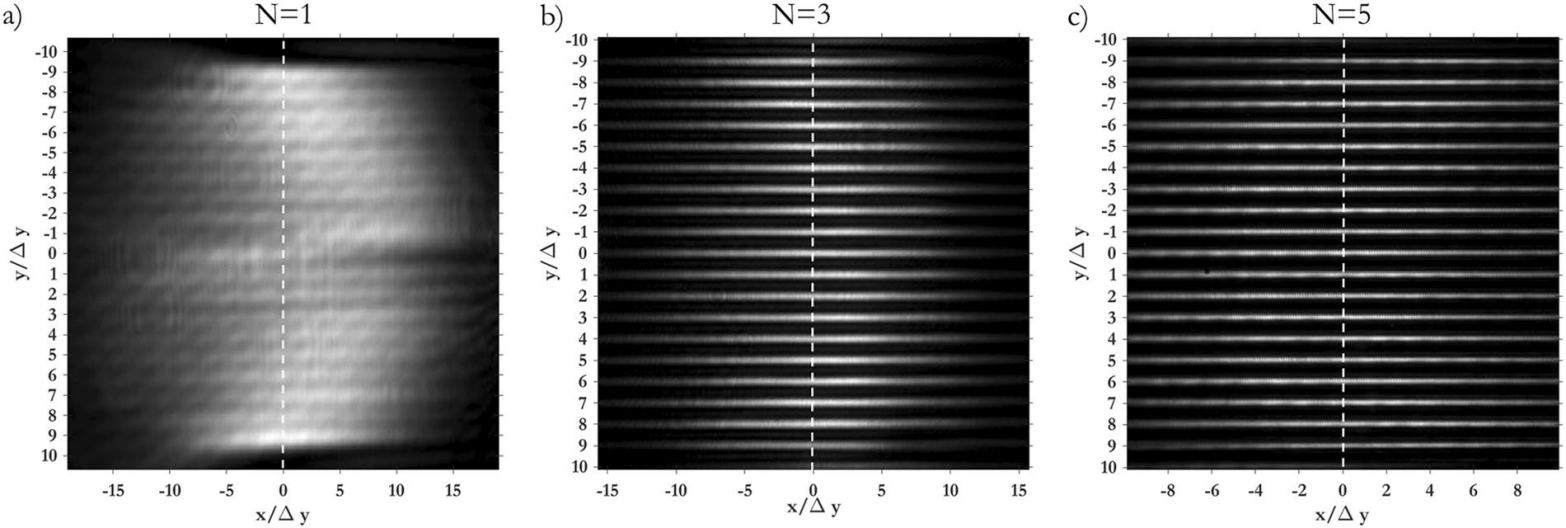
Experimental data for OAM beams in the range *ℓ* = −{9, …, +9} focused after the fan-out *log-pol* sorter for (**a**) *N* = 1 (**b**), *N* = 3 and (**c**), *N* = 5. As expected, a reduction in spot width is evident as the fan-out multiplication factor *N* increases. Coordinates have been normalized by the parameter Δ*y* = *λf*/2π*a*, such that the vertical position of each spot corresponds to the OAM content of the corresponding beam, according to Eq. (2).

**Figure 6:**
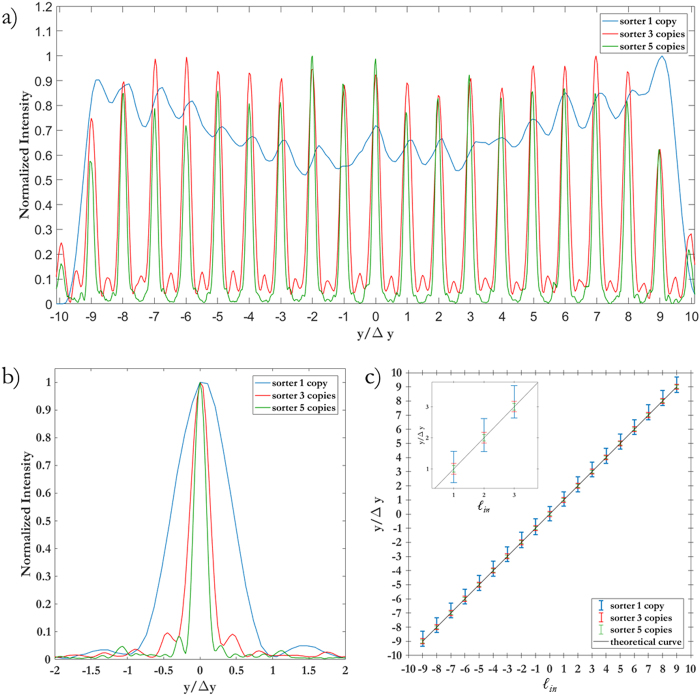
(**a**) Cross-sections of the experimental far-field plots in Fig. 5. The vertical coordinate has been normalized by Δ*y* = *λf*/2π*a*. (**b**) Intensity profile of the single peak corresponding to *ℓ* = 0. (**c**) Position of the spots as a function of the input OAM value and theoretical curve.

**Figure 7:**
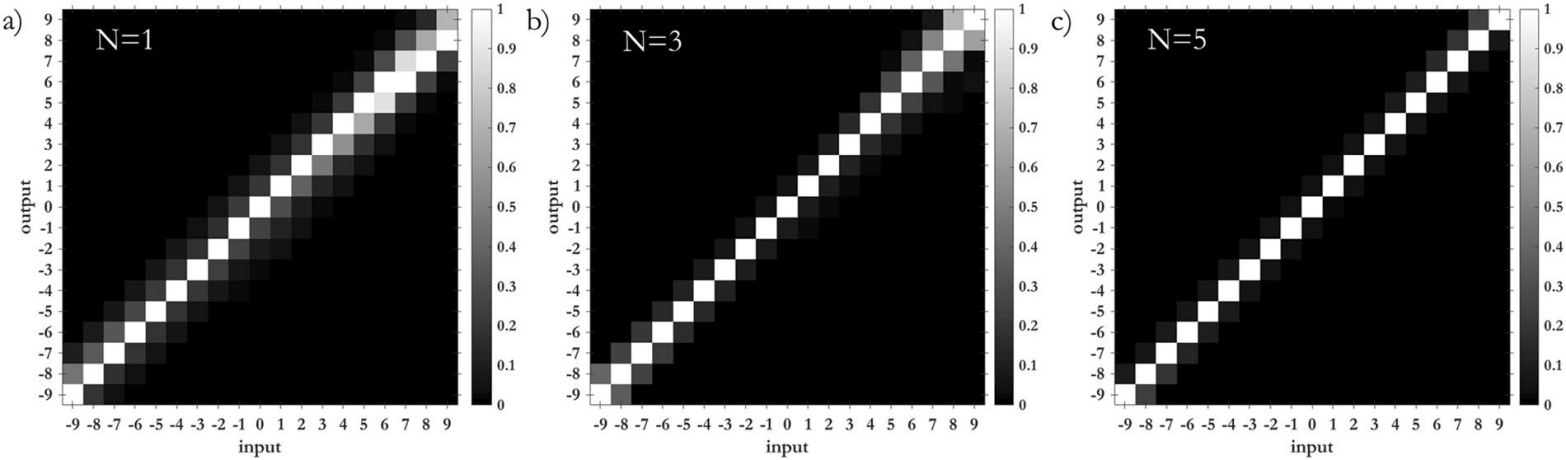
Output relative power in all detector regions for pure input OAM-beams in the set *ℓ* = {−9, …, +9}, for different number of copies *N* of the integrated fan-out *log-pol* sorter: (**a**) *N* = 1, (**b**) *N* = 3, (**c**) *N* = 5.

**Figure 8:**
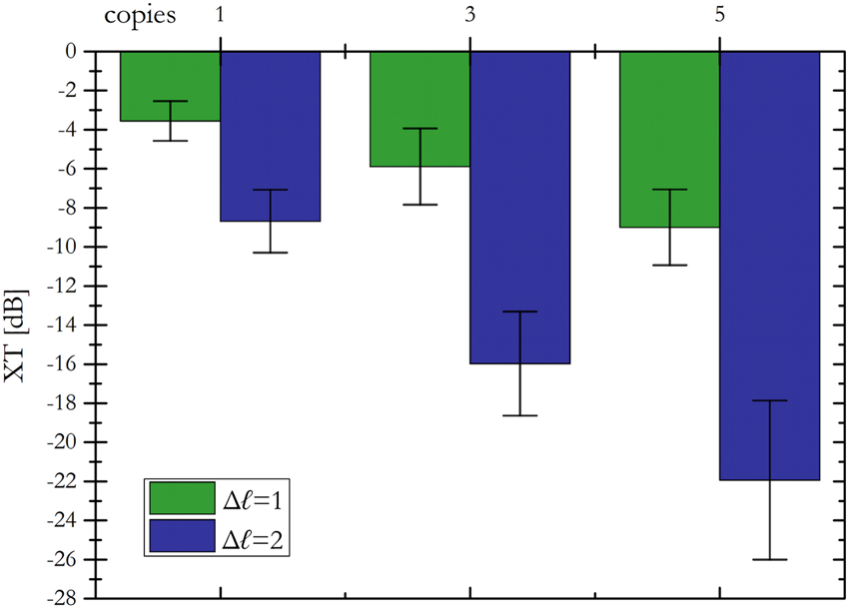
Average inter-channel cross-talk *XT* for the fan-out *log-pol* sorter as a function of copies number *N* = {1, 3, 5} and channel separation Δ*ℓ* = {1, 2}. Error bars: standard deviation calculated over the total channel set in the range *ℓ* = {−9, …, +9}.

As Figs 5 and 6 show, the width of the focused spots in far-field decreases with the number of copies generated by the fan-out term, as expected. While in the classical sorter consecutive modes are not separated (Fig. 5(a)) and part of the energy is collected by the neighbouring channels (Fig. 7(a)), the integration of the fan-out remarkably improves the resolution of the system. Consecutive OAM modes become clearly separated (Figs 5(b,c), and 6(a)), since the width of the output spots is reduced (Fig. 6(b)). As a consequence, the intensity of off-diagonal terms in the efficiency plots becomes lower (Fig. 7) and the cross-talk of the system is significantly improved (Fig. 8): *XT*(*N* = 1, Δ*ℓ* = 1) = −3.56 ± 1.02 dB, *XT*(*N* = 3, Δ*ℓ* = 1) = −5.89 ± 1.95 dB, *XT*(*N* = 5, Δ*ℓ* = 1) = −9.01 ± 1.94 dB. A further remarkable improvement can be obtained by increasing OAM separation. Limiting to Δ*ℓ* = 2, we get: *XT*(*N* = 1, Δ*ℓ* = 2) = −8.69 ± 1.61 dB, *XT*(*N* = 3, Δ*ℓ* = 2) = −15.98 ± 2.66 dB, *XT*(*N* = 5, Δ*ℓ* = 2) = −21.94 ± 4.07 dB.

**Figure 9:**
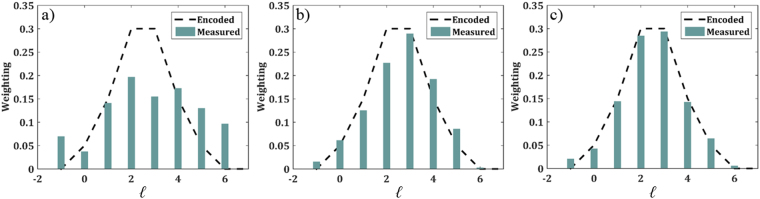
Encoded (multiplexed) and measured (de-multiplexed) superposition weightings for multiplexed OAM modes sent through (**a**) *N* = 1, (**b**) *N* = 3 and (**c**) *N* = 5 integrated copies of the fan-out *log-pol* mode sorter. The similarity between the multiplexed and de-multiplexed detected modes are *S* = 0.791, *S* = 0.968 and *S* = 0.971 for the cases of *N* = 1, 3 and 5, respectively.

Furthermore, analysis of Fig. 9 shows a distinct improvement on the accuracy of multimode OAM beam detection. A superposition of eight OAM modes in the Laguerre-Gaussian basis was created on the SLM, with a modal weighting as shown in the “encoded” plots of Fig. 9. The mode sorters were used to de-multiplex the modes and recover the weighting. We observe a large deviation between the encoded and detected weightings for the conventional sorter, Fig. 9(a), as a result of the increased cross-talk and associated interference between overlapping adjacent modes. This can be seen with the detected weightings of the outer OAM modes in the multiplexed array being significantly smaller than the encoded value while the detected weightings of the modes central to the array have a distinct increase, such that, overall, inaccurate values are detected. A large improvement is clear with the fan-out integration, Fig. 9(b,c), where the relative detected weightings agree well with the encoded values, reaching a similarity (discrete correlation) of over 97%.

**Figure 10:**
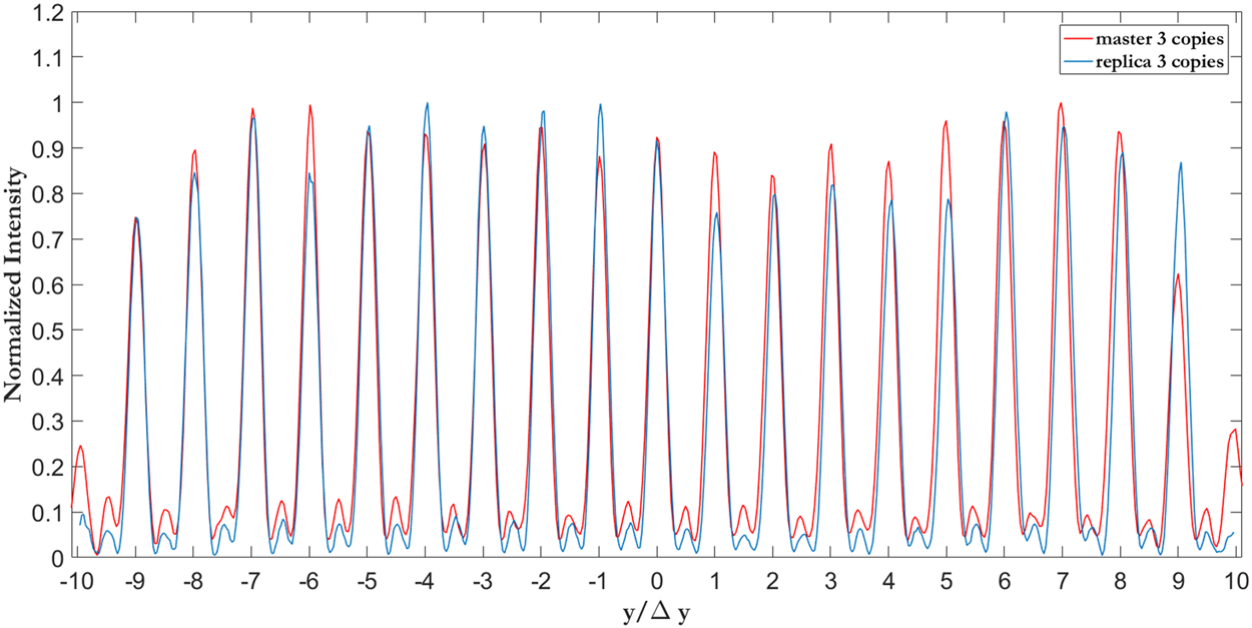
Comparison between the experimental cross-section of the far field distribution of the master and replica for the sorter with a three-copies fan-out.

The same optical characterization was performed on the sorters fabricated with soft-lithography replica process from the EBL masters. The generated replica reproduces the optical behaviour of the original sample with high fidelity, as clearly showed in Fig. 10 for the 3-copy sorter. In addition, it is worth noting that the background noise is lower in the replicated sample than in the master. This provides an improvement of the average cross-talk with a value down to *XT*(*N* = 3, Δ*ℓ* = 1) = −10.26 ± 1.78. This improvement is ascribed to the thermal smoothing of the sample surface after the imprinting process that reduces the roughness with respect to the master, with a consequent drop in scattering losses.

## Discussion

We designed and fabricated a compact and high-resolution sorter of optical beams carrying orbital angular momentum, by combining the well-known method based on conformal mapping with a fan-out optical operation. The unitary optical transformation is traditionally performed by means of two optical elements, i.e. unwrapper and phase-corrector, which efficiently convert the azimuthal phase gradients of OAM beams into linear phase gradients, then focused at different positions with a Fourier lens. However, due to the inherent diffraction process, neighbouring modes overlap one to each other, which dramatically limits the capability of the system to separate consecutive OAM states. A solution to the problem consists in spatially extending the linear phase gradients, in order to obtain, on the focal plane of the Fourier lens, narrower spots focused at the same positions as before. This was originally achieved by inserting, in sequence to the sorter, a fan-out element creating multiple copies of the beam followed by the corresponding phase-corrector, for a total of at least four elements plus lenses for Fourier transforms.

In this work, the original setup was compacted into two optical elements: a first one, performing both *log-pol* optical transformation and fan-out, followed by a double phase-corrector. The resulting optical device is markedly simplified and miniaturized as compared to previous demonstrations, while improving sorting performance. Moreover, incorporating the two elements into a single optical platform considerably reduced the alignment difficulty. We fabricated and tested the compact sorters with integrated *N*-copies fan-out for *N* = {1, 3, 5}. Electron-beam lithography provided a high-resolution method for the fabrication of the optical elements in the form of high-quality 3D phase-only diffractive optics. The optical performance of the samples has been characterized in terms of efficiency in OAM-mode separation and modal cross-talk. The integration of the fan-out in the sorting system provides a reduction in width of the output spots, therefore decreasing the overlap between neighbouring modes and improving the accuracy in OAM beam detection, as experimentally demonstrated. Cross-talk values down to −10 dB can be achieved without using sparse mode sets as in previous studies^21,22^. Values below −20 dB can be obtained increasing the OAM separation up to 2, even lower than the cross-talk provided by the standard sorter with a channel separation of 4^22^.

In addition, since electron-beam lithography, while extremely precise, is expensive in terms of time and costs, we considered the possibility to replicate the fabricated optics with faster mass-production methods, in the specific nano-imprinting. To this intent, we optimized a fabrication process for master replica made of UV-cured photopolymers, allowing higher throughput and much lower production costs. The generated replica reproduced the optical behaviour of the original sample with high fidelity, showing even better performance in efficiency and cross-talk.

To conclude, we experimentally demonstrated the possibility to increase the resolution of the sorter based on *log-pol* conformal mapping by integrating an optical fan-out. The fabrication technique and the new optical architecture remarkably improve the miniaturization level and the compactness of the system and therefore make feasible its integration and industrialisation. This work paves the way for practical OAM multiplexing and demultiplexing devices for use both in classical and quantum communication.

## Methods

### Numerical simulations

A custom MATLAB code was implemented, based on the convolution algorithm in the angular spectrum regime, in order to compute the propagation of a Gaussian beam impinging on the fan-out unwrapper and therefore calculate the phase pattern of the corresponding dual phase-corrector for phase-distortion correction. The same code was used to calculate the numerical output of the designed diffractive elements and estimate the OAM bandwidth of the sorter on the basis of the design parameters, e.g. *log-pol* transformation parameters (*a*, *b*) and focal length *f* of the system.

### Electron-beam lithography

All the diffractive optics have been fabricated by patterning a layer of PMMA resist (thickness of 2 µm, molecular weight of 950 kg/mol), spin-coated on a 1.1 mm thick ITO coated soda lime float glass substrate and pre-baked for 10 min at 180 °C on a hot plate. The low surface resistivity (8–12 Ω) of the ITO ensures a good discharge of the sample during lithography. From contact profilometry, the dose for a complete removal of the PMMA layer was found to be 566 µC/cm^2^. The phase patterns were written with a JBX-6300FS JEOL EBL machine, 12 MHz, generating at 100 KeV and 100 pA an electron-beam with a diameter of 2 nm, assuring a resolution down to 5 nm. Few nanometers of gold were sputtered on the surface of the transparent sample to ensure both a better determination of the beam focus and to improve the electron discharge during the exposure. Next, samples were developed for 60 s in a temperature-controlled bath (deionized water: isopropyl alcohol (IPA) 3:7) set on a magnetic stirrer at 1000 rpm. This specific developer was found to be the most suitable choice, providing optimized contrast characteristics and sensitivity as well as minimal surface roughness at the working temperature of 20 °C. After development, the optical elements were gently rinsed in deionized water and blow-dried under nitrogen flux.

### Soft-lithography replica

A suitable amount ofthe elastomer Sylgard 184 polydimethylsiloxane (PDMS)base was mixed with the catalyst in a weight ratio of 10:1 respectively, stirred thoroughly, and then cast onto the surface of the EBL fabricated master. The container was placed in a desiccator for30–45 minutes to de‐gas it and remove the trapped air bubbles formed during PDMS pouring and mixing. The sunken master with PDMS prepolymer was then placed into an oven at the temperature of 110 °C to be thermally cured for 30 minutes, then it was left in a freezer for 10 minutes to cool down. This process shrinks the PDMS slightly and helps when peeling the samples out of their molds. In order to fabricate the replica of the mold, a UV-curable photopolymer (Norland Optical Adhesive 74) was dropped onto a glass substrate. The PDMS mold was overlaid on it (with the patterned side facing the liquid) and pressed lightly to form the contact and evacuate the air bubbles trapped under the mold. Different UV-exposure times have been tested in order to fine tune the optimal recipe for best replica production (refer to Supplementary information for further details). The optical performance of the fabricated sorter replica was examined considering sorting efficiency and inter-channel cross-talk, and a UV-curing time of 240 s has been experimentally demonstrated to be the best choice. Afterwards, the PDMS mold was carefully peeled off to get the final replica.

### Optical Characterization

The Gaussian beam (*λ* = 632.8 nm, beam waist *w*_0_ = 240 μm, power 0.8 mW) emitted by a HeNe laser source (HNR008R, Thorlabs) is linearly polarized (LPVISE100-A, Thorlabs) before illuminating the display of a LCoS spatial light modulator (PLUTO-NIR-010-A, Holoeye, 1920 × 1080 pixels, 8 μm pixel size, 8-bit depth), combining axicon and spiral phase functions with a quadratic term for curvature correction. The beam reflected by the SLM is Fourier-transformed with a first lens L1 of focal length *f*_1_ = 40 cm. Then a 50:50 beam-splitter is used to split the beam and analyze field profile and OAM content at the same time. The field profile is collected with a CMOS camera (DCC1545M, Thorlabs, 1280 × 1024 pixels, 5.2 μm pixel size, monochrome, 8-bit depth). The beam illuminates the first outer zone of the sorter sample, mounted on a 6-axis kinematic mount (K6XS, Thorlabs. Kinematic pitch/yaw: ±4° at 5 mrad/rev, X and Y translation: ±2 mm at 254 µm/rev, continuous 360° goniometer with 1° graduations) and located at the focus of lens L1. A mirror is placed on a kinematic mount (KM100, Thorlabs) and the position is controlled with a micrometric translator (TADC-651, Optosigma). The distance from the sorter is equal to half the focal length *f* of the unwrapper term. After passing through the sorter inner zone, the signal is collected by a second CCD camera (1500M-GE, Thorlabs, 1392 × 1040 pixels, 6.45 μm pixel size, monochrome, 12-bit depth) placed at the back-focal plane of a lens with *f*_2_ = 12.5 cm.

Characterization of the sorter multiplexing ability was accomplished through expansion and collimation of a horizontally polarized HeNe laser beam (*λ* = 632.8 nm, power 2 mW) onto a phase-only SLM (PLUTO-VIS HoloEye, 8 µm, 1920 × 1080 pixels) with a 10x objective lens and 30.0 cm lens. An *f-f* system with an aperture in the Fourier plane (FP) isolated and imaged the first order encoded mode onto the first sorting optic. Both sorting optics were mounted on tip-tilt rotational kinematic and translational mounts with a separation distance equal to the design incorporated focal length in the unwrapping term. A 10.0 cm lens was placed *f*-away from the second, phase-correcting term. The sorted spots formed in the FP were then captured with a Spiricon SP620u CCD camera. Laguerre-Gaussian beam (*w*_0_ = 600 μm) superpositions were generated through phase and amplitude modulation with a variation of weightings as indicated by the theoretical black lines in Fig. 9.

## Electronic supplementary material

Supplementary information
