# Supplementary material for: A compact diffractive sorter for high-resolution demultiplexing of orbital angular momentum beams
Source: Sci Rep. 2018 Jul 6;8:10248. doi: 10.1038/s41598-018-28447-1 (PMC6035274; doi:10.1038/s41598-018-28447-1)
Supplement: Supplementary file 1 — Supplementary information [file 41598_2018_28447_MOESM1_ESM.pdf]

Supplementary information

A compact diffractive sorter for high-resolution demultiplexing of orbital angular momentum beams

Gianluca Ruffato[1,2], Marcello Girardi [1,2], Michele Massari[1,2], Erfan Mafakheri[1,2], Bereneice Sephton[3], Pietro Capaldo[2,4], Andrew Forbes[3] and Filippo Romanato[1,2,3*]

[1]Department of Physics and Astronomy ‘G. Galilei’, University of Padova, via Marzolo 8, 35131 Padova, Italy

[2]LaNN, Laboratory for Nanofabrication of Nanodevices, EcamRicert, Corso Stati Uniti 4, 35127 Padova, Italy.

[3]School of Physics, University of the Witwatersrand, Private Bag 3, Wits, South Africa

[4]CNR-INFM TASC IOM National Laboratory, S.S. 14 Km 163.5, 34012 Basovizza, Trieste, Italy

[*]Corresponding author: filippo.romanato@unipd.it

S1. SOFT-LITHOGRAPHY PROCESS OPTIMIZATION

During the first tests of the replica process the replicas showed an increase in the background noise, i.e. the scattered radiation, if compared with the master. This effect has been attributed to some deviations in the replica process, since the original masters exhibited better values of crosstalk. Since the PDMS mold was made following a standard process [1], the imperfect curing of the resin has been considered and investigated.

The average cross-talk has been chosen as control parameter for each replica, therefore a set of OAM beams in the range from -6 to +6 have been sorted for each sample, and the same data analysis of the master has been repeated. The sample chosen was a three-copies fan-out sorter. The choice was driven by the aligning time of the sample on the optical table.

The radiation dose has been changed by increasing the exposure time of the NOA sample under UV light, and the curve in Supplementary Figure 1 has been obtained. There is an almost abrupt discontinuity between the underexposed replica and the samples with an optimal curing time. The first four doses (30-90-120-160 s) show a crosstalk around -5.5 dB, far from the value obtained with the master. It is noticeable that with an exposure time of 200 s the crosstalk starts to decrease, and it drops to -8.71±1.12 dB with a curing time of 240 s. After 240 s the crosstalk slowly increases, suggesting either a mild degradation of the mold or a thermal damage of the resin, due to the impinging radiation. However the fluctuation is minimal and within the error bars of the average cross-talk. Supplementary Figure 2 shows SEM inspections of two replicas of the 3-copy sorters fabricated at different UV-curing times. The choice of 240 s confirms the better definition of the surface-relief profile, especially in correspondence of phase discontinuities, and the higher smoothness of the surface.

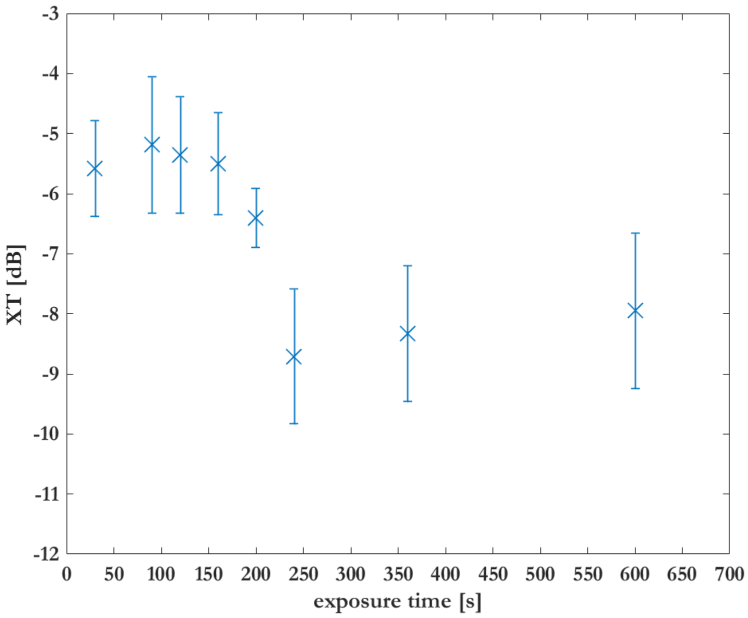

Supplementary Figure 1. Average cross-talk of the 3-copies fan-out sorter replica as a function of the UV-curing time.

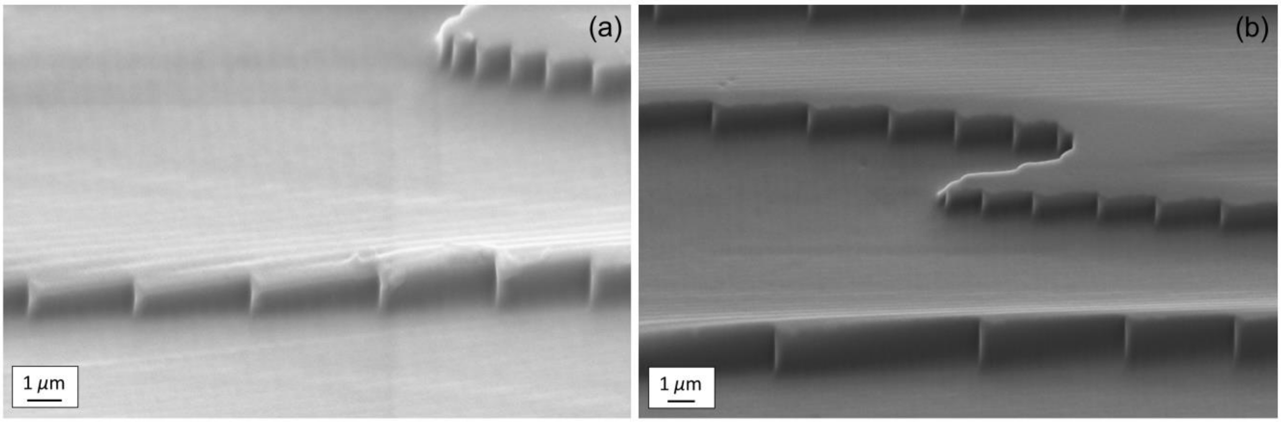

Supplementary Figure 2. SEM inspection of two 3-copy sorter replica fabricated at different UV-curing time: 30 s (a), 240 s (b).

S2. OPTICAL CHARACTERISATION

The experimental setup used for characterization of the mode-sorter (MS) elements is given in Supplementary Figure 3(a). The same setup was used to study the performance of the sorters with regards to sorting multiplexed OAM modes. In this analysis, the fan-out unwrapper and the double phase-corrector were written on two separated glass slides. A HeNe beam (633nm) was expanded with an objective lens and collimated to overfill a spatial light modulator (SLM) used to generate vortex beams through phase and amplitude modulation. The 1st order was then isolated, demagnified by a factor of 2 and imaged onto the first mode sorter element (unwrapper) through a 4-*f* telescope system with an aperture in the Fourier plane. Images of the unwrapper and phase-corrector elements can be seen for the 1-copy case in Suppl. Fig. 3(b).

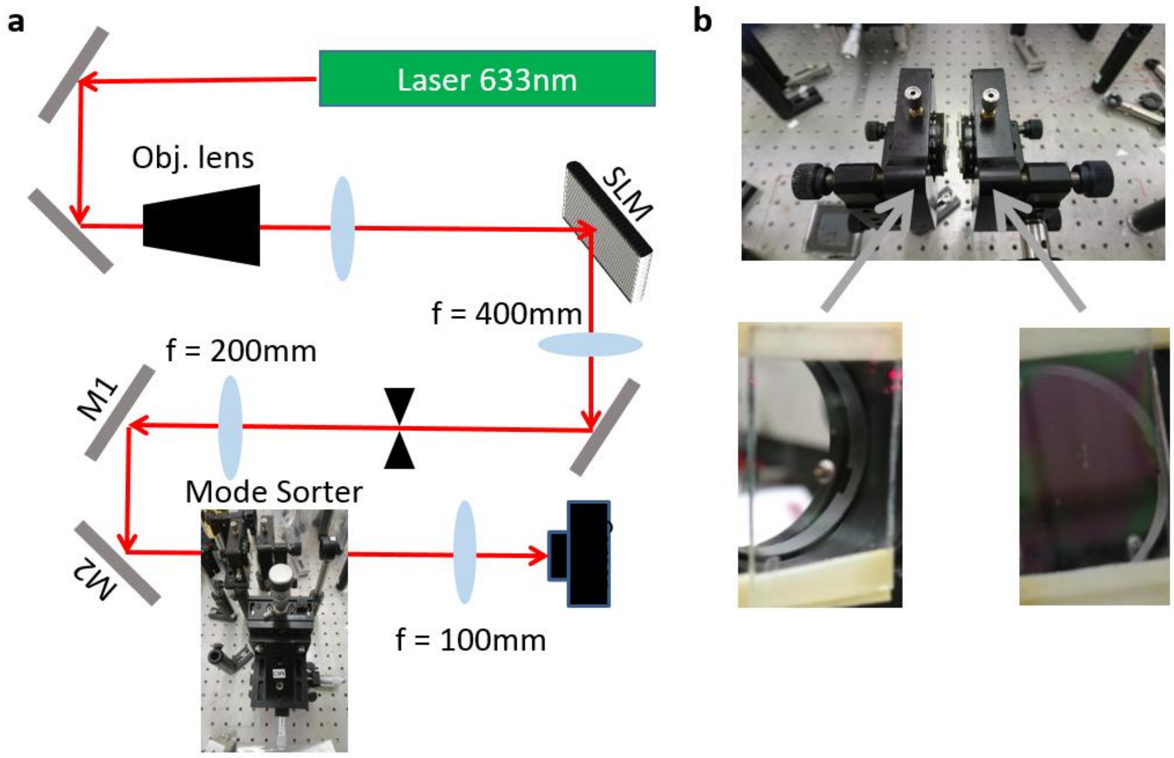

Supplementary Figure 3. (a) Simplified experimental setup used to characterize the diffractive optics mode sorters and (b) close-up of holders used to align the mode sorter elements as well as the transformation areas of the MS elements themselves.

Due to the sensitivity of mode sorters to alignment, the 1st element was mounted with double-sided tape onto a holder allowing for *x*-and *y*-transverse plane adjustment as well as tip-tilt degrees of freedom. Two opposing mirrors were included before the mode sorter so as to allow for walking the beam through the elements and thus allowing for easy control of the input beam. The second element requires an additional degree of freedom (DoF) in the *z*-axis or optical axis direction as the separation distance requires a great degree of precision in addition to the minute separation distance of 8.500 mm being difficult to position without the control afforded by a translation stage. The second element was thus also attached onto the same type of mount with double sided tape and that attached to a *x*-,*y*- and *z*-translation stage as depicted in Suppl. Fig. 3(a). Varying the *z*-axis DoF also allows for alignment to be streamlined. For the 5-copy mode sorter case, the additional copies increased the associated sensitivity of the alignment requiring translations stages that were more stable than for the previous examples.

A 100 mm lens was used after the MS to transform the unraveled and phase-corrected beam into OAM-dependent lateral positions in a Fourier lens configuration (*f*-*f* configuration) and a CCD placed at the focal plane. A short focal length was required as the output beam diverges quickly after the 2nd element.

The positions of the OAM modes were determined theoretically and experimentally. A plot of the actual measured spot positions against that which is theoretically calculated is given between OAM = [-15, 15] in Suppl. Fig. 4 for the 1-and 3-copy sorters and between [-5,5] for the 5-copy sorter.

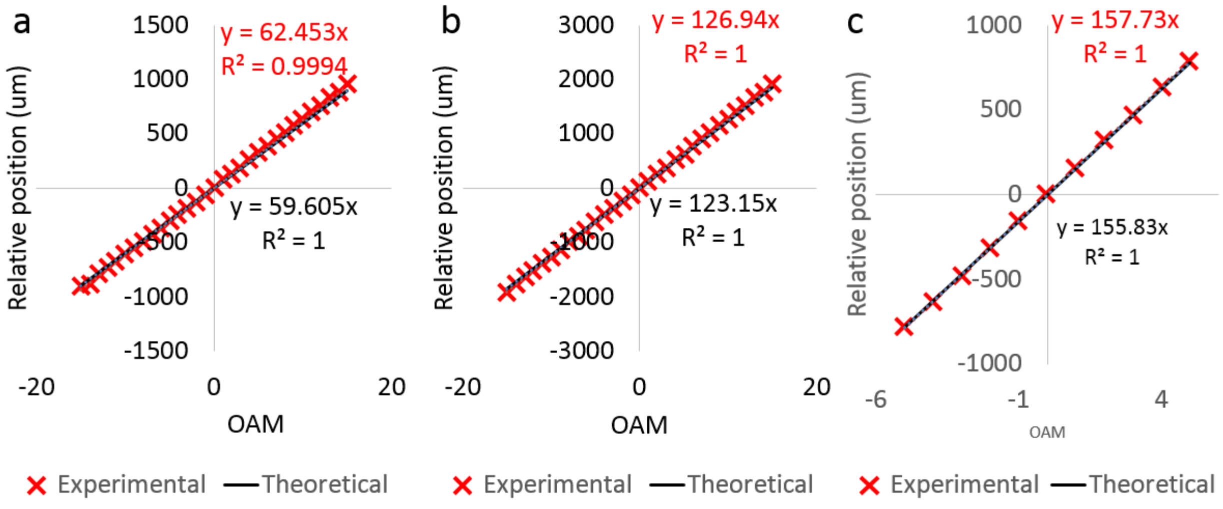

Supplementary Figure 4. A comparison of measured (red) sorted spot positions against that which was theoretically expected (black) based on the experimentally measured unraveled beam length for the (a) 1-copy, (b) 3-copy and (c) 5-copy mode sorter.

For given wavelength and focal length, the theoretical separation distance Δ*s* is determined by the MS design parameter *a*, according to $\Delta s = \lambda f/(2\pi a)$. The expected value was calculated to be 63 μm compared to an average experimental value of 62.5 μm (red line gradient) in Suppl. Fig. 4(a) for the 1-copy mode sorter. For the 3-copy mode sorter, the theoretical separation distance was calculated to be 126.5 μm which compared to an average experimental value of 126.9 μm (red line gradient) is in excellent agreement. Similarly, good agreement was seen for the 5-copy sorter where an experimental average separation of 157.7 μm can be seen in comparison to the theoretical separation distance of 155.8 μm based on the measured unwrapped beam length. Values are reported in Supplementary Table 1.

Sorter	$2\pi a$ (μm)	Δs_{theo} (μm)	Δs_{exp} (μm)
1-copy MS	1000	63.0	62.5
3-copy MS	500	126.5	126.9
5-copy MS	400	155.8	157.7

Supplementary Table 1. Theoretical (Δs_{theo}) and experimental (Δs_{exp}) channel separation for the fabricated 1-, 3- and 5-copy sorters.

S3. SIMILARITY

The comparative accuracy of the multiplexed and detected OAM modes was determine through its similarity as given by:

$$S = \frac{\left[\sum_{\ell} \sqrt{W_{\exp}(\ell) W_{theo}(\ell)}\right]^2}{\sum_{\ell} W_{\exp}(\ell) \sum_{\ell} W_{theo}(\ell)}$$

where $W_{theo}(\ell)$ is the theoretical or multiplexed weighting associated with the OAM mode ℓ and $W_{exp}(\ell)$ is the detected equivalent of the mode. A significant increase in the accuracy of modal detection occurs as the copy numbers increase. More specifically, the similarity of 79.1% for the 1-copy is increased by 22% when adding 2-copies which is further refined to a 97.1% similarity when 5-copy is utilized.

REFERENCES

[1] Johnston, I. D., McCluskey D. K., Tan, C. K. L., and Tracey, M. C. Mechanical characterization of bulk Sylgard 184 for microfluidics and microengineering. *J. Micromech. Microeng.* **24**, 035017 (2014).
